# Supplementary material for: Transcriptome Analysis Reveals Vimentin-Induced Disruption of Cell–Cell Associations Augments Breast Cancer Cell Migration
Source: Cells. 2022 Dec 13;11(24):4035. doi: 10.3390/cells11244035 (PMC9776984; doi:10.3390/cells11244035)
Supplement: Supplementary file 1 [file cells-11-04035-s001.zip › DEGs and enrichment analysis.zip!/DEGs and enrichment analysis/WT_VIMvsVector KEGG/GSEA/WT_VIM/MICRORNAS_IN_CANCER/MICRORNAS_IN_CANCER(HSA05206).html]

Details for gene set MICRORNAS\_IN\_CANCER(HSA05206)[GSEA]

|  || Dataset | fpkm.sample |
| Phenotype | sample.cls |
| Upregulated in class | WT\_VIM |
| GeneSet | MICRORNAS\_IN\_CANCER(HSA05206) |
| Enrichment Score (ES) | 0.8437081 |
| Normalized Enrichment Score (NES) | 1.4010241 |
| Nominal p-value | 0.0 |
| FDR q-value | 0.16499993 |
| FWER p-Value | 0.0 |
Table: GSEA Results Summary

  

Fig 1: Enrichment plot: MICRORNAS\_IN\_CANCER(HSA05206)      
 Profile of the Running ES Score & Positions of GeneSet Members on the Rank Ordered List

  

| PROBE | DESCRIPTION (from dataset) | GENE SYMBOL | GENE\_TITLE | RANK IN GENE LIST | RANK METRIC SCORE | RUNNING ES | CORE ENRICHMENT || 1 | ENSG00000237026 | AC104073.2 |  |  | 10 | Infinity | -0.0002 | Yes |
| 2 | ENSG00000259207 | ITGB3 |  |  | 391 | Infinity | -0.0066 | Yes |
| 3 | ENSG00000231300 | EZH2P1 |  |  | 724 | Infinity | -0.0122 | Yes |
| 4 | ENSG00000250662 | HNRNPKP5 |  |  | 1540 | Infinity | -0.0261 | Yes |
| 5 | ENSG00000116147 | TNR |  |  | 1918 | Infinity | -0.0325 | Yes |
| 6 | ENSG00000238247 | AC006210.2 |  |  | 2255 | Infinity | -0.0382 | Yes |
| 7 | ENSG00000225602 | MTOR-AS1 |  |  | 2354 | Infinity | -0.0398 | Yes |
| 8 | ENSG00000026025 | VIM |  |  | 2388 | 1709.539 | 0.8397 | Yes |
| 9 | novel.507 | - |  |  | 2498 | 11.391 | 0.8437 | Yes |
| 10 | ENSG00000174775 | HRAS |  |  | 3007 | 4.878 | 0.8376 | No |
| 11 | ENSG00000171791 | BCL2 |  |  | 3339 | 3.945 | 0.8340 | No |
| 12 | ENSG00000230626 | AC011005.1 |  |  | 3357 | 3.863 | 0.8357 | No |
| 13 | ENSG00000101412 | E2F1 |  |  | 3381 | 3.812 | 0.8373 | No |
| 14 | ENSG00000243064 | ABCC13 |  |  | 3437 | 3.716 | 0.8383 | No |
| 15 | ENSG00000283480 | AL512380.2 |  |  | 3438 | 3.716 | 0.8402 | No |
| 16 | ENSG00000227347 | HNRNPKP2 |  |  | 3645 | 3.435 | 0.8384 | No |
| 17 | ENSG00000164045 | CDC25A |  |  | 3735 | 3.317 | 0.8386 | No |
| 18 | ENSG00000185634 | SHC4 |  |  | 3775 | 3.302 | 0.8397 | No |
| 19 | ENSG00000156103 | MMP16 |  |  | 3809 | 3.267 | 0.8408 | No |
| 20 | ENSG00000068078 | FGFR3 |  |  | 3893 | 3.145 | 0.8410 | No |
| 21 | ENSG00000146670 | CDCA5 |  |  | 3900 | 3.138 | 0.8425 | No |
| 22 | ENSG00000148400 | NOTCH1 |  |  | 4161 | 2.890 | 0.8396 | No |
| 23 | ENSG00000026508 | CD44 |  |  | 4248 | 2.820 | 0.8396 | No |
| 24 | ENSG00000169047 | IRS1 |  |  | 4281 | 2.798 | 0.8405 | No |
| 25 | ENSG00000088305 | DNMT3B |  |  | 4325 | 2.786 | 0.8412 | No |
| 26 | ENSG00000101224 | CDC25B |  |  | 4808 | 2.486 | 0.8343 | No |
| 27 | ENSG00000154342 | WNT3A |  |  | 4840 | 2.472 | 0.8350 | No |
| 28 | ENSG00000158402 | CDC25C |  |  | 5024 | 2.391 | 0.8331 | No |
| 29 | ENSG00000130816 | DNMT1 |  |  | 5048 | 2.377 | 0.8340 | No |
| 30 | ENSG00000100985 | MMP9 |  |  | 5081 | 2.361 | 0.8347 | No |
| 31 | ENSG00000130669 | PAK4 |  |  | 5481 | 2.207 | 0.8290 | No |
| 32 | novel.453 | - |  |  | 6009 | 2.031 | 0.8211 | No |
| 33 | ENSG00000126934 | MAP2K2 |  |  | 6037 | 2.023 | 0.8217 | No |
| 34 | ENSG00000117632 | STMN1 |  |  | 6448 | 1.935 | 0.8157 | No |
| 35 | ENSG00000250859 | HNRNPKP1 |  |  | 6568 | 1.909 | 0.8147 | No |
| 36 | ENSG00000135423 | GLS2 |  |  | 6629 | 1.893 | 0.8146 | No |
| 37 | ENSG00000012048 | BRCA1 |  |  | 6637 | 1.891 | 0.8155 | No |
| 38 | ENSG00000102882 | MAPK3 |  |  | 6737 | 1.867 | 0.8148 | No |
| 39 | ENSG00000185338 | SOCS1 |  |  | 6847 | 1.855 | 0.8139 | No |
| 40 | ENSG00000143590 | EFNA3 |  |  | 6938 | 1.831 | 0.8133 | No |
| 41 | ENSG00000136997 | MYC |  |  | 7014 | 1.815 | 0.8130 | No |
| 42 | ENSG00000137801 | THBS1 |  |  | 7054 | 1.804 | 0.8132 | No |
| 43 | ENSG00000235852 | AC005540.1 |  |  | 7256 | 1.761 | 0.8107 | No |
| 44 | ENSG00000159217 | IGF2BP1 |  |  | 7426 | 1.730 | 0.8087 | No |
| 45 | ENSG00000075618 | FSCN1 |  |  | 7475 | 1.720 | 0.8088 | No |
| 46 | ENSG00000137807 | KIF23 |  |  | 7571 | 1.701 | 0.8081 | No |
| 47 | ENSG00000137193 | PIM1 |  |  | 7596 | 1.695 | 0.8085 | No |
| 48 | ENSG00000226648 | PLCG1-AS1 |  |  | 7719 | 1.673 | 0.8073 | No |
| 49 | ENSG00000139514 | SLC7A1 |  |  | 8034 | 1.618 | 0.8028 | No |
| 50 | ENSG00000149418 | ST14 |  |  | 8061 | 1.614 | 0.8032 | No |
| 51 | ENSG00000145675 | PIK3R1 |  |  | 8151 | 1.601 | 0.8025 | No |
| 52 | ENSG00000074181 | NOTCH3 |  |  | 8196 | 1.595 | 0.8026 | No |
| 53 | ENSG00000108840 | HDAC5 |  |  | 8230 | 1.588 | 0.8029 | No |
| 54 | ENSG00000141564 | RPTOR |  |  | 8354 | 1.568 | 0.8016 | No |
| 55 | ENSG00000175305 | CCNE2 |  |  | 8401 | 1.561 | 0.8016 | No |
| 56 | ENSG00000167193 | CRK |  |  | 8813 | 1.506 | 0.7954 | No |
| 57 | ENSG00000204188 | GGNBP1 |  |  | 8912 | 1.494 | 0.7945 | No |
| 58 | ENSG00000115266 | APC2 |  |  | 9135 | 1.462 | 0.7915 | No |
| 59 | ENSG00000124181 | PLCG1 |  |  | 9178 | 1.457 | 0.7915 | No |
| 60 | ENSG00000134853 | PDGFRA |  |  | 9324 | 1.439 | 0.7898 | No |
| 61 | ENSG00000134250 | NOTCH2 |  |  | 9332 | 1.438 | 0.7904 | No |
| 62 | ENSG00000099942 | CRKL |  |  | 9349 | 1.437 | 0.7909 | No |
| 63 | ENSG00000141510 | TP53 |  |  | 9380 | 1.433 | 0.7911 | No |
| 64 | ENSG00000111276 | CDKN1B |  |  | 9723 | 1.392 | 0.7860 | No |
| 65 | ENSG00000160691 | SHC1 |  |  | 9802 | 1.382 | 0.7854 | No |
| 66 | ENSG00000198793 | MTOR |  |  | 10025 | 1.356 | 0.7823 | No |
| 67 | ENSG00000105976 | MET |  |  | 10079 | 1.349 | 0.7821 | No |
| 68 | ENSG00000005339 | CREBBP |  |  | 10148 | 1.340 | 0.7817 | No |
| 69 | ENSG00000166484 | MAPK7 |  |  | 10159 | 1.339 | 0.7822 | No |
| 70 | ENSG00000184349 | EFNA5 |  |  | 10193 | 1.335 | 0.7823 | No |
| 71 | ENSG00000185950 | IRS2 |  |  | 10335 | 1.322 | 0.7806 | No |
| 72 | ENSG00000243364 | EFNA4 |  |  | 10601 | 1.305 | 0.7768 | No |
| 73 | ENSG00000099617 | EFNA2 |  |  | 10639 | 1.300 | 0.7768 | No |
| 74 | ENSG00000030110 | BAK1 |  |  | 10737 | 1.290 | 0.7758 | No |
| 75 | ENSG00000097007 | ABL1 |  |  | 10991 | 1.266 | 0.7722 | No |
| 76 | ENSG00000171608 | PIK3CD |  |  | 11010 | 1.264 | 0.7725 | No |
| 77 | ENSG00000243547 | HNRNPKP4 |  |  | 11315 | 1.236 | 0.7680 | No |
| 78 | ENSG00000224057 | EGFR-AS1 |  |  | 11463 | 1.230 | 0.7661 | No |
| 79 | ENSG00000175730 | BAK1P1 |  |  | 11604 | 1.219 | 0.7644 | No |
| 80 | ENSG00000068024 | HDAC4 |  |  | 11711 | 1.209 | 0.7632 | No |
| 81 | ENSG00000068028 | RASSF1 |  |  | 11806 | 1.200 | 0.7622 | No |
| 82 | ENSG00000169032 | MAP2K1 |  |  | 11855 | 1.195 | 0.7620 | No |
| 83 | ENSG00000177885 | GRB2 |  |  | 11958 | 1.185 | 0.7609 | No |
| 84 | ENSG00000100784 | RPS6KA5 |  |  | 12180 | 1.166 | 0.7578 | No |
| 85 | ENSG00000100393 | EP300 |  |  | 12306 | 1.156 | 0.7562 | No |
| 86 | ENSG00000106462 | EZH2 |  |  | 12485 | 1.142 | 0.7538 | No |
| 87 | ENSG00000126583 | PRKCG |  |  | 12565 | 1.135 | 0.7531 | No |
| 88 | ENSG00000116478 | HDAC1 |  |  | 12725 | 1.122 | 0.7509 | No |
| 89 | ENSG00000197943 | PLCG2 |  |  | 12777 | 1.118 | 0.7506 | No |
| 90 | ENSG00000100311 | PDGFB |  |  | 12913 | 1.107 | 0.7489 | No |
| 91 | ENSG00000136158 | SPRY2 |  |  | 13138 | 1.092 | 0.7457 | No |
| 92 | ENSG00000204044 | SLC12A5-AS1 |  |  | 13210 | 1.087 | 0.7450 | No |
| 93 | ENSG00000158715 | SLC45A3 |  |  | 13211 | 1.087 | 0.7456 | No |
| 94 | ENSG00000153094 | BCL2L11 |  |  | 13248 | 1.083 | 0.7455 | No |
| 95 | ENSG00000132155 | RAF1 |  |  | 13338 | 1.077 | 0.7446 | No |
| 96 | ENSG00000110092 | CCND1 |  |  | 13381 | 1.074 | 0.7444 | No |
| 97 | ENSG00000261351 | AC116913.1 |  |  | 14173 | 1.013 | 0.7315 | No |
| 98 | ENSG00000103275 | UBE2I |  |  | 14299 | 1.003 | 0.7299 | No |
| 99 | ENSG00000065361 | ERBB3 |  |  | 14906 | 0.976 | 0.7201 | No |
| 100 | ENSG00000165119 | HNRNPK |  |  | 14914 | 0.975 | 0.7205 | No |
| 101 | ENSG00000254013 | MAP2K1P1 |  |  | 15216 | 0.958 | 0.7159 | No |
| 102 | ENSG00000197461 | PDGFA |  |  | 15245 | 0.956 | 0.7159 | No |
| 103 | ENSG00000261505 | AL031714.1 |  |  | 15280 | 0.953 | 0.7158 | No |
| 104 | ENSG00000169242 | EFNA1 |  |  | 15334 | 0.950 | 0.7154 | No |
| 105 | ENSG00000112242 | E2F3 |  |  | 15451 | 0.942 | 0.7139 | No |
| 106 | ENSG00000138061 | CYP1B1 |  |  | 15537 | 0.937 | 0.7129 | No |
| 107 | ENSG00000100030 | MAPK1 |  |  | 15645 | 0.929 | 0.7116 | No |
| 108 | ENSG00000213281 | NRAS |  |  | 15905 | 0.926 | 0.7077 | No |
| 109 | novel.446 | - |  |  | 16128 | 0.911 | 0.7044 | No |
| 110 | ENSG00000067560 | RHOA |  |  | 16287 | 0.900 | 0.7022 | No |
| 111 | ENSG00000196591 | HDAC2 |  |  | 16306 | 0.899 | 0.7023 | No |
| 112 | ENSG00000150593 | PDCD4 |  |  | 16568 | 0.884 | 0.6983 | No |
| 113 | ENSG00000171862 | PTEN |  |  | 16769 | 0.871 | 0.6954 | No |
| 114 | ENSG00000105173 | CCNE1 |  |  | 17069 | 0.852 | 0.6908 | No |
| 115 | ENSG00000141736 | ERBB2 |  |  | 17129 | 0.849 | 0.6902 | No |
| 116 | ENSG00000168209 | DDIT4 |  |  | 17161 | 0.847 | 0.6901 | No |
| 117 | novel.264 | - |  |  | 17371 | 0.835 | 0.6870 | No |
| 118 | ENSG00000113328 | CCNG1 |  |  | 17451 | 0.830 | 0.6861 | No |
| 119 | ENSG00000206557 | TRIM71 |  |  | 17452 | 0.830 | 0.6865 | No |
| 120 | novel.398 | - |  |  | 17606 | 0.821 | 0.6843 | No |
| 121 | ENSG00000164305 | CASP3 |  |  | 17672 | 0.818 | 0.6836 | No |
| 122 | ENSG00000147889 | CDKN2A |  |  | 17982 | 0.800 | 0.6788 | No |
| 123 | ENSG00000171132 | PRKCE |  |  | 18248 | 0.785 | 0.6747 | No |
| 124 | ENSG00000119772 | DNMT3A |  |  | 18420 | 0.775 | 0.6722 | No |
| 125 | ENSG00000096717 | SIRT1 |  |  | 18650 | 0.762 | 0.6687 | No |
| 126 | ENSG00000105647 | PIK3R2 |  |  | 18727 | 0.758 | 0.6678 | No |
| 127 | ENSG00000249506 | ZEB2P1 |  |  | 18840 | 0.752 | 0.6663 | No |
| 128 | ENSG00000104365 | IKBKB |  |  | 18846 | 0.751 | 0.6666 | No |
| 129 | ENSG00000124762 | CDKN1A |  |  | 19129 | 0.735 | 0.6622 | No |
| 130 | ENSG00000122707 | RECK |  |  | 19211 | 0.732 | 0.6612 | No |
| 131 | ENSG00000109320 | NFKB1 |  |  | 19286 | 0.727 | 0.6603 | No |
| 132 | ENSG00000112715 | VEGFA |  |  | 19350 | 0.723 | 0.6596 | No |
| 133 | ENSG00000103222 | ABCC1 |  |  | 19381 | 0.721 | 0.6595 | No |
| 134 | ENSG00000118971 | CCND2 |  |  | 19488 | 0.716 | 0.6580 | No |
| 135 | ENSG00000092820 | EZR |  |  | 19495 | 0.716 | 0.6583 | No |
| 136 | ENSG00000133703 | KRAS |  |  | 19512 | 0.715 | 0.6584 | No |
| 137 | ENSG00000149311 | ATM |  |  | 19523 | 0.714 | 0.6586 | No |
| 138 | ENSG00000104081 | BMF |  |  | 19656 | 0.707 | 0.6567 | No |
| 139 | ENSG00000067900 | ROCK1 |  |  | 19799 | 0.700 | 0.6547 | No |
| 140 | ENSG00000198625 | MDM4 |  |  | 19828 | 0.698 | 0.6546 | No |
| 141 | ENSG00000168283 | BMI1 |  |  | 19910 | 0.693 | 0.6535 | No |
| 142 | ENSG00000204217 | BMPR2 |  |  | 20445 | 0.664 | 0.6448 | No |
| 143 | ENSG00000267317 | AC027307.2 |  |  | 20588 | 0.656 | 0.6427 | No |
| 144 | ENSG00000114861 | FOXP1 |  |  | 20703 | 0.650 | 0.6411 | No |
| 145 | ENSG00000140416 | TPM1 |  |  | 20795 | 0.646 | 0.6399 | No |
| 146 | ENSG00000100292 | HMOX1 |  |  | 20858 | 0.643 | 0.6392 | No |
| 147 | ENSG00000100697 | DICER1 |  |  | 20930 | 0.639 | 0.6383 | No |
| 148 | ENSG00000117461 | PIK3R3 |  |  | 21106 | 0.632 | 0.6357 | No |
| 149 | ENSG00000121879 | PIK3CA |  |  | 21520 | 0.608 | 0.6290 | No |
| 150 | ENSG00000148516 | ZEB1 |  |  | 21641 | 0.600 | 0.6273 | No |
| 151 | ENSG00000134982 | APC |  |  | 21669 | 0.597 | 0.6271 | No |
| 152 | ENSG00000105810 | CDK6 |  |  | 21736 | 0.593 | 0.6263 | No |
| 153 | ENSG00000135679 | MDM2 |  |  | 21791 | 0.590 | 0.6257 | No |
| 154 | ENSG00000104290 | FZD3 |  |  | 21909 | 0.582 | 0.6240 | No |
| 155 | ENSG00000161638 | ITGA5 |  |  | 21997 | 0.578 | 0.6228 | No |
| 156 | ENSG00000143384 | MCL1 |  |  | 22351 | 0.554 | 0.6171 | No |
| 157 | ENSG00000115904 | SOS1 |  |  | 22567 | 0.539 | 0.6137 | No |
| 158 | ENSG00000168610 | STAT3 |  |  | 22678 | 0.531 | 0.6121 | No |
| 159 | ENSG00000051382 | PIK3CB |  |  | 22747 | 0.525 | 0.6112 | No |
| 160 | ENSG00000124766 | SOX4 |  |  | 22767 | 0.524 | 0.6112 | No |
| 161 | ENSG00000229124 | VIM-AS1 |  |  | 22784 | 0.523 | 0.6112 | No |
| 162 | ENSG00000168477 | TNXB |  |  | 22816 | 0.520 | 0.6109 | No |
| 163 | ENSG00000137710 | RDX |  |  | 23239 | 0.492 | 0.6040 | No |
| 164 | ENSG00000225066 | Z93929.1 |  |  | 23329 | 0.486 | 0.6028 | No |
| 165 | ENSG00000113721 | PDGFRB |  |  | 23668 | 0.464 | 0.5973 | No |
| 166 | ENSG00000100485 | SOS2 |  |  | 23862 | 0.450 | 0.5942 | No |
| 167 | ENSG00000154229 | PRKCA |  |  | 23896 | 0.447 | 0.5939 | No |
| 168 | ENSG00000120332 | TNN |  |  | 24097 | 0.429 | 0.5907 | No |
| 169 | ENSG00000237984 | PTENP1 |  |  | 24128 | 0.427 | 0.5904 | No |
| 170 | ENSG00000115419 | GLS |  |  | 24140 | 0.426 | 0.5904 | No |
| 171 | ENSG00000073756 | PTGS2 |  |  | 24256 | 0.418 | 0.5887 | No |
| 172 | ENSG00000263006 | ROCK1P1 |  |  | 24562 | 0.394 | 0.5837 | No |
| 173 | ENSG00000146648 | EGFR |  |  | 24583 | 0.392 | 0.5836 | No |
| 174 | ENSG00000276058 | STMN1P1 |  |  | 24721 | 0.381 | 0.5815 | No |
| 175 | ENSG00000073282 | TP63 |  |  | 24927 | 0.361 | 0.5782 | No |
| 176 | ENSG00000100234 | TIMP3 |  |  | 25006 | 0.352 | 0.5770 | No |
| 177 | ENSG00000019186 | CYP24A1 |  |  | 25028 | 0.349 | 0.5769 | No |
| 178 | ENSG00000203435 | E2F3P2 |  |  | 25666 | 0.286 | 0.5662 | No |
| 179 | ENSG00000085563 | ABCB1 |  |  | 26122 | 0.238 | 0.5586 | No |
| 180 | ENSG00000267691 | SHC1P2 |  |  | 26281 | 0.218 | 0.5560 | No |
| 181 | ENSG00000277443 | MARCKS |  |  | 26297 | 0.216 | 0.5559 | No |
| 182 | ENSG00000122861 | PLAU |  |  | 26311 | 0.215 | 0.5558 | No |
| 183 | ENSG00000092969 | TGFB2 |  |  | 26493 | 0.192 | 0.5528 | No |
| 184 | ENSG00000222047 | C10orf55 |  |  | 26546 | 0.186 | 0.5520 | No |
| 185 | ENSG00000206075 | SERPINB5 |  |  | 26667 | 0.166 | 0.5500 | No |
| 186 | ENSG00000275741 | AL162497.1 |  |  | 27460 | 0.000 | 0.5366 | No |
| 187 | ENSG00000169554 | ZEB2 |  |  | 27993 | 0.000 | 0.5276 | No |
| 188 | ENSG00000232754 | AL035658.1 |  |  | 28129 | 0.000 | 0.5253 | No |
| 189 | ENSG00000041982 | TNC |  |  | 28637 | 0.000 | 0.5167 | No |
| 190 | ENSG00000268173 | AC007192.1 |  |  | 29070 | 0.000 | 0.5093 | No |
| 191 | ENSG00000128710 | HOXD10 |  |  | 29294 | 0.000 | 0.5055 | No |
| 192 | ENSG00000169946 | ZFPM2 |  |  | 29362 | 0.000 | 0.5044 | No |
| 193 | ENSG00000007968 | E2F2 |  |  | 29891 | 0.000 | 0.4954 | No |
| 194 | ENSG00000262313 | AC127496.3 |  |  | 30049 | 0.000 | 0.4928 | No |
| 195 | ENSG00000235196 | Z68868.1 |  |  | 31064 | NaN | 0.4756 | No |
| 196 | ENSG00000218991 | CCNG1P1 |  |  | 31737 | NaN | 0.4641 | No |
| 197 | ENSG00000276915 | AL109618.3 |  |  | 32041 | NaN | 0.4590 | No |
| 198 | ENSG00000219867 | AL590635.1 |  |  | 32117 | NaN | 0.4577 | No |
| 199 | ENSG00000273860 | AC024581.2 |  |  | 33080 | NaN | 0.4414 | No |
| 200 | novel.443 | - |  |  | 33169 | NaN | 0.4399 | No |
| 201 | ENSG00000230994 | FGFR3P1 |  |  | 33426 | NaN | 0.4356 | No |
| 202 | ENSG00000226488 | LINC01824 |  |  | 33954 | NaN | 0.4266 | No |
| 203 | ENSG00000249943 | AC097486.1 |  |  | 34061 | NaN | 0.4248 | No |
| 204 | ENSG00000269897 | COMMD3-BMI1 |  |  | 34282 | NaN | 0.4211 | No |
| 205 | ENSG00000281358 | RASSF1-AS1 |  |  | 35038 | NaN | 0.4083 | No |
| 206 | ENSG00000267046 | E2F3P1 |  |  | 35277 | NaN | 0.4043 | No |
| 207 | ENSG00000225943 | AC016907.1 |  |  | 35302 | NaN | 0.4039 | No |
| 208 | ENSG00000243635 | AC091804.1 |  |  | 36423 | NaN | 0.3848 | No |
| 209 | ENSG00000234724 | HDAC1P1 |  |  | 36859 | NaN | 0.3775 | No |
| 210 | ENSG00000256674 | AC020549.1 |  |  | 37130 | NaN | 0.3729 | No |
| 211 | ENSG00000236616 | BAK1P2 |  |  | 38612 | NaN | 0.3477 | No |
| 212 | ENSG00000232418 | AC074348.1 |  |  | 39926 | NaN | 0.3254 | No |
| 213 | ENSG00000261882 | AC006600.1 |  |  | 40122 | NaN | 0.3221 | No |
| 214 | ENSG00000233995 | AP000547.1 |  |  | 40567 | NaN | 0.3146 | No |
| 215 | ENSG00000251440 | STMN1P2 |  |  | 40775 | NaN | 0.3111 | No |
| 216 | ENSG00000269926 | DDIT4-AS1 |  |  | 40851 | NaN | 0.3098 | No |
| 217 | ENSG00000204301 | NOTCH4 |  |  | 41377 | NaN | 0.3009 | No |
| 218 | ENSG00000224071 | AC009474.1 |  |  | 41749 | NaN | 0.2946 | No |
| 219 | ENSG00000277241 | AC026355.3 |  |  | 42186 | NaN | 0.2872 | No |
| 220 | ENSG00000273600 | AP001877.1 |  |  | 42303 | NaN | 0.2853 | No |
| 221 | ENSG00000230889 | SHC1P1 |  |  | 42363 | NaN | 0.2843 | No |
| 222 | ENSG00000256847 | CCND2P1 |  |  | 44079 | NaN | 0.2551 | No |
| 223 | ENSG00000231610 | PIK3CDP1 |  |  | 44917 | NaN | 0.2409 | No |
| 224 | ENSG00000223691 | AC068044.1 |  |  | 45579 | NaN | 0.2297 | No |
| 225 | ENSG00000248290 | TNXA |  |  | 45919 | NaN | 0.2240 | No |
| 226 | ENSG00000237033 | CASP3P1 |  |  | 45940 | NaN | 0.2236 | No |
| 227 | ENSG00000255387 | AC015689.2 |  |  | 46104 | NaN | 0.2209 | No |
| 228 | ENSG00000234201 | BMI1P1 |  |  | 46641 | NaN | 0.2118 | No |
| 229 | ENSG00000251246 | AL691442.1 |  |  | 46799 | NaN | 0.2091 | No |
| 230 | ENSG00000220635 | KRASP1 |  |  | 47607 | NaN | 0.1954 | No |
| 231 | ENSG00000233012 | HDAC1P2 |  |  | 48183 | NaN | 0.1857 | No |
| 232 | ENSG00000234961 | AL133415.1 |  |  | 48561 | NaN | 0.1793 | No |
| 233 | ENSG00000226446 | NOTCH2P1 |  |  | 48628 | NaN | 0.1781 | No |
| 234 | ENSG00000261559 | FSCN1P1 |  |  | 49873 | NaN | 0.1570 | No |
| 235 | ENSG00000220548 | VIM2P |  |  | 50683 | NaN | 0.1433 | No |
| 236 | ENSG00000223391 | AC006210.1 |  |  | 51395 | NaN | 0.1312 | No |
| 237 | ENSG00000166501 | PRKCB |  |  | 52822 | NaN | 0.1070 | No |
| 238 | ENSG00000250040 | RAF1P1 |  |  | 52866 | NaN | 0.1063 | No |
| 239 | ENSG00000255920 | CCND2-AS1 |  |  | 54034 | NaN | 0.0865 | No |
| 240 | ENSG00000262067 | AC120057.1 |  |  | 54826 | NaN | 0.0731 | No |
| 241 | ENSG00000108379 | WNT3 |  |  | 54890 | NaN | 0.0720 | No |
| 242 | ENSG00000282278 | AC058822.1 |  |  | 54950 | NaN | 0.0710 | No |
| 243 | ENSG00000260984 | FGFR3P5 |  |  | 56034 | NaN | 0.0526 | No |
| 244 | ENSG00000259753 | AC068234.1 |  |  | 56362 | NaN | 0.0471 | No |
| 245 | ENSG00000278621 | AC037198.2 |  |  | 57243 | NaN | 0.0321 | No |
Table: GSEA details [plain text format]

  

Fig 2: MICRORNAS\_IN\_CANCER(HSA05206)      
 Blue-Pink O' Gram in the Space of the Analyzed GeneSet

  

Fig 3: MICRORNAS\_IN\_CANCER(HSA05206): Random ES distribution      
 Gene set null distribution of ES for **MICRORNAS\_IN\_CANCER(HSA05206)**
